# REIMAGINING ALZHEIMER’S RESEARCH: ON THE ROAD TO NEW DISCOVERIES

**DOI:** 10.1093/geroni/igac059.1584

**Published:** 2022-12-20

**Authors:** Stacy Andersen, Lenora Smith, Allison Gibson

## Abstract

Organized by the Alzheimer's disease and related dementias (ADRD) Interest Group, this symposium aims to highlight unique approaches to address 1) ADRD risk reductions through social and biological factors, and 2) quality of life of those with dementia. The first two presentations introduce remote communication technologies used for behavioral interventions, one for preventing social isolation and the resultant cognitive decline using video-chats by recruiting socially isolated older adults with mild cognitive impairment (MCI). The results of the recently completed randomized controlled trial provide evidence of a positive effect on cognition suggesting enhanced cognitive reserve. Another is for delivering a behavioral symptom intervention for community-dwelling persons with ADRD using telehealth. Researchers will highlight the feasibility, opportunities, and challenges of remote behavioral interventions. The third presentation focuses on micro-level communication between persons living with dementia and their family caregivers. By examining video-recorded home care observations, findings suggest caregiver education on positive interactions could facilitate more meaningful interaction with persons with ADRD. The fourth presentation explores pain among those with ADRD, a multifaceted phenomenon with health and functional consequences. Using the NHATS dataset, researchers show a continued need to detect and address pain in clinical settings particularly among persons with ADRD. The symposium concludes with a presentation using the CHARLS study to investigate correlations between inflammation or kidney-related metabolic biomarkers and cognition. Results provide longitudinal evidence of an association of kidney function and inflammation with cognitive test scores supporting the need to broaden dementia etiology research beyond the amyloid-cascade theory.

